# "The Hospital" Medical Book Supplement—No XXXII

**Published:** 1910-08-20

**Authors:** 


### "THE HOSPITAL" MEDICAL BOOK SUPPLEMENT—No XXXII.

CONTENTS.

| | Page |
|---|---|
| **Medicine—** | |
| Squire's Pharmacopœias of the London Hospitals ... ... | 621 |
| Lectures on Cosmetic Treatment ... ... ... ... ... ... | 621 |
| Address on the Conquest of Consumption and the Duties of the Municipalities ... ... ... ... ... ... ... ... | 621 |
| Mentally Deficient Children: their Treatment and Training ... | 622 |
| Physiology, the Servant of Medicine ... ... ... ... ... | 622 |
| **Midwifery—** | |
| The Practice of Midwifery ... ... ... ... ... ... ... ... | 622 |
| Difficult Labour ... ... ... ... ... ... ... ... ... ... | 623 |
| **Surgery—** | |
| Movable Kidney, its Etiology, Pathology, Diagnosis, Symptoms, and Treatment ... ... ... ... ... ... ... ... | 623 |
| **Miscellaneous—** | |
| The Bacteriologist's Aid to Memory ... ... ... ... ... | 624 |
| The Preservation of the Hair ... ... ... ... ... ... ... | 624 |
| A Handbook for Nurses ... ... ... ... ... ... ... ... | 624 |
| Dogs, their Breeds and Characteristics ... ... ... ... | 624 |
| Advice to Consumptives: Home Treatment, After-care, and Prevention ... ... ... ... ... ... ... ... ... ... | 624 |
| Human Anatomical Model ... ... ... ... ... ... ... | 624 |

### MEDICINE.

Squire's Pharmacopœias of the London Hospitals. (London: J. and A. Churchill, 7 Great Marlborough Street. Price 5s. net.)

This book is a comparison of the Pharmacopœias of thirty London hospitals, with the Pharmacopœias of the Children's Hospitals and the German Hospital as addenda. The first edition of the "London Hospitals Pharmacopœia," was published by the late Peter Squire in 1863, so that for nearly half a century this little book has been a recognised work of reference to the medical profession. It was an extension of the comparison of the three Pharmacopœias of London, Edinburgh, and Dublin from which the first edition of "Squire's Companion to the British Pharmacopœia" was evolved. The seventh edition was published in 1900, and it is a noteworthy fact that in the subsequent ten years, no fewer than twenty-six of the London Hospitals have produced new editions. So numerous and extensive have been the alterations in the formulæ that this eighth edition had to be practically rewritten. At the same time, many new formulæ have been introduced. The new eighth edition would have been produced earlier but for the delay necessitated by the enormous amount of literary matter which had to be dealt with in editing the recently published eighteenth edition of "Squire's Companion to the British Pharmacopœia"; the postponement was therefore unavoidable, although the seventh edition of "Squire's London Hospitals" has been out of print for two years. It will be found invaluable to members of the medical profession, as it represents select methods of prescribing very many drugs, and forms a practical compendium of prescriptions framed by the leading authorities in the profession. A leaflet descriptive of the book, which reproduces typical specimen pages and briefly reviews its aims and objects, will be forwarded gratis to those applying for it to Squire and Sons, Chemists on the Establishment of his Majesty the King, 413 Oxford Street, London, W.

Lectures on Cosmetic Treatment. By Dr. Edmund Saalfeld. Translated by J. F. Halls Dally, M.A., M.D., B.C. Cantab. (London: Rebman, Ltd. 1910. Price 5s. net.)

Some time ago the author delivered a series of lectures on cosmetic treatment to medical men, and he has now embodied these lectures in the present volume. Cosmetics form undoubtedly a branch of dermatology, and it is most unsatisfactory to think that dermatology is taught in its broad sense, yet never by any chance is there any instruction given to the student in what is really a most important branch of skin diseases—namely, cosmetic treatment. What is more annoying to anyone, especially a woman, than to suffer from some form of acne, or to be a martyr to "freckles," and yet to know that the ordinary doctor will merely say that "it is nothing to worry over, and that in time it will go away of its own accord," simply because he happens to know but little of cosmetic healing? Small wonder that these patients drift into the hands of mountebanks who set up as skin specialists and beauty doctors. Dr. Saalfeld's book is a thoroughly good little volume, contains many useful prescriptions, and will prove a blessing to any practitioner who will consult it when faced by some fair patient who would be cured of some slight, though disfiguring, skin affection, which to her is a serious matter.

Address on the Conquest of Consumption and the Duties of the Municipalities. By Arthur Latham, M.D., F.R.C.P. New Tract Series. No. 8. Manchester and Salford Sanitary Association. (London: Sherratt and Hughes. 1910. Price 1d.)

Starting with the statement that consumption is not hereditary, and that increased opportunity of infection explains the apparent superiority in numbers of the affected offspring of tuberculous parents as compared with those of healthy parents, the author plunges at once into his subject, and rapidly reviews the preventive and curative measures which should be employed to overcome a disease which is annually responsible for the deaths of 60,000 persons in these islands; or, as he remarks, more than any great war has cost us. He is a firm believer in the value of anti-tuberculosis dispensaries. Members of the working classes can go for advice when their work is over, no money is lost as at a hospital by the loss of half a day's work, and cases can be detected in their early and curable stage and a watch kept over them. Sanatorium treatment can then be carried out in suitable cases, and provision made for their dependents. Unfortunately the present accommodation available for those needing sanatorium treatment would only serve to deal with about 1½ per cent. of the cases. The author, however, points out that it has been actuarially calculated that for ½d. a week paid into a central fund by every member of the community over fifteen years of age, accommodation can be provided for all in need of it, and that for an additional 2½ millions a year provision can be made for dependents. The present cost to the nation of the disease is about 8 millions a year, paid by friendly societies, the Poor Law, and special hospitals, and yet practically nothing is effected as regards prevention and cure. The author is a supporter of tuberculin treatment, coupled with that of the sanatorium. He concludes that with proper preventive measures, and the provision of sufficient dispensaries and adequate sanatorium accommodation—*i.e.* for 60,000 patients a year—the disease would be stamped out in twenty or thirty years.

Mentally Deficient Children: Their Treatment and Training. By G. E. Shuttleworth, B.A., M.D., etc., and W. A. Potts, B.A., M.D., etc. Third Edition. (London: H. K. Lewis. 1910. Crown 8vo. Price 5s. net.)

In view of the daily increasing interest evinced by the public in all that appertains to the welfare of children, the appearance of a third edition of this work is welcome. It should be the aim of every practitioner to have at least a superficial knowledge of what differentiates mental deficiency from certifiable lunacy, and a volume such as the present will be found extremely useful as an introduction to the subject. After a brief historical retrospect regarding previous efforts in many countries to deal with the mentally deficient, the authors plunge at once into their subject and classify the forms the condition may take. This is followed by a description of the ætiology, diagnosis, and prognosis of each, with suggestions as to the medical examination of children requiring special instruction, and hints as to the treatment of each form of deficient mentality. Treatment in addition to general, is educational and moral. The authors betray no undue enthusiasm as to the results to be obtained. They acknowledge that cures are rare, though a few undoubtedly occur. The best that can be hoped for in most cases is an improvement such as will enable the patient, if not to keep himself, at least to contribute substantially to his own support. The authors regret that the steps so far taken to deal with the mentally deficient have been too sporadic to be generally useful. The permissive legislation which allows of school authorities establishing residential schools for epileptics has been ineffective and should be replaced by compulsory legislation. There can be no doubt that good has resulted from the Defective and Epileptic Children Act of 1899, but it is by no means a solution of the problem. Moreover, provision should be made for the adequate supervision, and if necessary boarding out, of patients who have passed school age. The text of the book is illustrated by well-chosen photographs of the various types of mentally afflicted children.

Physiology, the Servant of Medicine. By Augustus D. Waller, M.D., LL.D., F.R.S. (London: The University of London Press and Hodder and Stoughton. 1910. Price 5s.)

As the author states in his preface, particular prominence is given in these lectures to the present day physiological knowledge of anæsthetics. They give on the purely scientific side the principal results of his own laboratory work during the past seven years, and are the continuation of two previous series of lectures on "Animal Electricity" (1897) and on the "Signs of Life" (1903). The author studies isolated muscle and nerve with reference to the effects on each respectively of various stimuli—alcohol, ether, chloroform, carbonic acid, hellebore, aconite, heat and light. He describes much that is new, and one or two things that are of practical value in medicine. His method of testing the electrical reaction of the skin before and after death leads to the hope that we are perhaps on the threshold of the discovery of a reliable test as to what constitutes death. At present only a negative sign shows that death is present—*i.e.* the absence of what the author terms a "blaze-current." Such currents have, however, been found in skin as long as fourteen days after death, so that death may be present although a "blaze-current" exists. The author's remarkable record with his method of administering a definite percentage of chloroform, by which he has anæsthetised some thousands of animals during the past six years without the accidental loss of a single one, would seem to be good ground for supposing that an equally satisfactory record might be obtained with it in the case of the human subject. He shows in his last lecture how the objections to the method could easily be overcome, and how, while not advising its use in the case of the country practitioner to whom the bulk of the apparatus as well as difficulties in obtaining suitable assistance, both mechanical and human, for its working would act as serious drawbacks, it could easily be used in hospital practice. The whole book is extremely interesting and written in the author's usual clear and pleasing style.

### MIDWIFERY.

The Practice of Midwifery. By Alfred Lewis Galabin, M.A., M.D. Cantab., F.R.C.P. Lond.; and George Blacker, M.D., B.S. Lond., F.R.C.S. Eng., F.R.C.P. Lond. (London J. and A. Churchill, 1910. Price 18s. net.)

Of all our systems of midwifery, perhaps none is so widely known as, or has been more extensively read than, that written by Dr. Galabin. In this, the latest edition, in which the author has collaborated with Dr. Blacker, the high standard of the book has been maintained to the full; in fact, if possible an advance has been made on its excellence, and we unhesitatingly say that the present issue of this justly popular work may be termed a classic on midwifery, for from beginning to end its teaching is of the soundest, and the practice it advocates has been bought at the expense of the long experience of its authors. The book has been enlarged to the extent of some two hundred pages, has been mostly rewritten, and contains chapters thoroughly up-to date on the development of the ovary and early ovum, the mechanism of labour, the pathology of eclampsia, accidental complications of pregnancy, etc. A new chapter has been added on "Injuries and Diseases of the Fœtus." One hundred and seventy-four new figures have been inserted, and in many other respects the book has been brought thoroughly up to date. One of the most useful chapters is that on the "Management of Normal Labour," and any man who is starting his medical career as a qualified individual could do no better than carefully read this section through, for if he knew it thoroughly it would save him many an anxious hour. It is a strange fact—yet none the less true—that a practitioner will often attend a woman in her confinement and examine her only when labour is about to commence. It is easy to see how absolutely incorrect such a practice is, and what dire results may follow through omitting to see the patient some weeks—if necessary months—before her confinement, and carefully measure the pelvis in its different diameters, and ascertain as early as possible the presentation of the child. If this rule were only followed much trouble would be saved both to the medical man and to the patient. It cannot be too greatly urged that the free use of forceps, so as to effect a speedy delivery, is a mistake. If Nature is but allowed to have her way, and given time, the birth of the child is easily brought about in most cases, and the comfort of the mother is better maintained during labour, and most certainly after. We do not mean to say that there are not many cases, apart from those in which forceps are absolutely necessary, in which instrumental delivery is helpful, but we do wish to disparage the free use of instruments in a perfectly *normal* labour. Another chapter that should prove invaluable is the one on "Accidental Complications of Pregnancy." We do not think it is fully understood what a very serious complication chronic cardiac disease may prove to be to a pregnant woman. Such cases require very careful watching, and if the patient goes to term it is all important to get the labour over quickly, for the obvious reason that in some cases the heart may give out any moment. Another complication that the authors treat most admirably is that of phthisis. They advise "That phthisical women should not marry, both on account of the increased risk to themselves, the probable phthisical predisposition of their children, and the possible communication of contagion to their husbands." The advice seems to us thoroughly sound, for we have frequently seen the effects of a woman who has had phthisis, both on herself and on her child; sometimes it has meant death to both, and in some cases death to the woman. There is an excellent chapter on "Version," and as this is one of those operations that can frequently be performed in general practice, and to which much kudos is attached, it is one which all medical men should carefully study. Cæsarean section, Symphysiotomy, and Pubiotomy are fully dealt with in another chapter, both from the operative standpoint and the after-treatment, as also are accidents during and after labour. Once again, in closing this review, we reiterate that the book is a classic fully worthy to find a place in the library of the general practitioner, since it gives sound and good advice on abstruse points of obstetrical practice and on the simpler ones of ordinary pregnancy.

Difficult Labour. By G. Ernest Herman, M.B. Lond., F.R.C.P., F.R.C.S. (London: Cassell and Co., Ltd. 1910. 12s. 6d.)

It would not be easy to find a medical man or student who did not know this work. At any London hospital, or provincial for that matter, it is a *sine qua non* that he who would pass his midwifery with ease must read and know his Herman's *Difficult Labour*; and indeed the author must think with pride of the thousands of students he has helped to pass their obstetrical examination, and to whom his book has been a perfect godsend! This new and enlarged edition outshines its predecessors. It appears in a new cover, it has many new illustrations, the whole work has been reset; finally, it contains two new sections, one on puerperal eclampsia, and the other on the effects and treatment of retroversion of the gravid uterus. One of the best chapters in it is that on "Abnormal Uterine Action," in which the difference between uterine inertia and tonic contraction of the uterus is clearly and fully dealt with. The author has had the happy idea of tabulating the chief points of variance, so that there is no excuse for anyone not to understand this most important phase of obstetrical practice. As the author points out, the difference in the treatment of the two conditions is completely opposite, for while in uterine inertia it is so important to make the patient sleep and delay delivery, in tonic contraction of the uterus it is all important to hasten delivery, even to the extent of crushing the head if necessary. Another chapter which is most excellent is that on post-partum hæmorrhage. This is eminently clear and lucid, and tells the student exactly what to do. Dr. Herman holds that drugs are useless in such cases, for they take a certain time in which to act, and in that time the patient dies. The only chance of saving the patient's life in some cases of bleeding is by continuous pressure, and it is just in explaining how to do this in the best possible way to produce that continuous pressure that Dr. Herman writes particularly clearly. The two new chapters that we have already mentioned are quite up to the high standard of others contained in this volume. In dealing with retroversion in pregnancy, the author wishes his readers thoroughly to grasp the point that "the sole importance of retroversion of the gravid uterus is that it sometimes causes retention of urine," and he makes this somewhat paradoxical statement—a statement which conveys to the mind at once the importance of, and thereby supplies a solution to, the whole question—"Displacement is nothing, incarceration everything. The uterus is nothing, the bladder everything." The treatment of the condition, it seems to us, hinges on that statement. The chapter on puerperal eclampsia is also excellent, and after discussing the ætiology, symptoms, and diagnosis of the condition, the author sums up briefly, but plainly, the treatment of these cases. We heartily congratulate Dr. Herman upon his new edition, which can be honestly recommended to students and practitioners alike, as perhaps the best manual on the subject with which it deals.

### SURGERY.

Movable Kidney, its Etiology, Pathology, Diagnosis, Symptoms, and Treatment. By William Billington, M.S. Lond., F.R.C.S. (London: Cassell and Co., Ltd. 1910. Price 7s. 6d. net.)

The author's extensive experience in operative measures for the fixation of displaced kidneys, as well as his unrivalled opportunities of gauging the evil effects of such conditions when left untreated, make his opinions worthy of respect. He has attempted in the present volume to set forth clearly and concisely the reasons which have led him to attach such great importance to the displaced kidney as the cause of neurasthenical, hysterical, and even diseased mental conditions. He would seem to have made out his case, for in the great majority of his patients the correction of the abnormal position of the organ by suitable abdominal supports or operative measures has led to either complete cure of, or at least marked improvement in, their symptoms. The sceptic will find it difficult to explain these facts otherwise than does the author. In addition to the nervous and mental disorders mentioned above, nephropexy would seem to be beneficial in several forms of functional derangement, such as chronic constipation, mucous colitis, flatulence, dysmenorrhœa, menorrhagia, etc., and it is curious to find that marked benefit has followed the operation in several cases of Raynaud's disease—associated with kidney mobility. Gibson, of Edinburgh, considers that in some cases the disease is caused by reflex irritation of the vasomotor system, due to a movable kidney. The chapters devoted to the anatomy of the kidney and to the description of the author's own method of operating are clear and well-expressed, and the illustrations and diagrams excellent.

### MISCELLANEOUS.

The Bacteriologist's Aid to Memory. By J. W. S. Seccombe, M.R.C.S., L.R.C.P., D.P.H. (London : John Bale, Sons and Danielsson, Limited. Price 3s. 6d. net.)

This "Aid to Memory" takes the form of a large tabulated chart, and the author calls it "a handy reference-sheet for the laboratory." Certainly he must have spent a considerable time in compiling it, and it must have been a most laborious task, but whether the time and labour spent upon it justifies the result is a question open to doubt. We cannot confidently recommend the chart to the general practitioner.

The Preservation of the Hair. By R. W. Leftwich, M.D. (London : Simpkin, Marshall, Hamilton, Kent and Co. 1910. Price 1s. net.)

This is a useful and practical little book, perhaps most useful to the hairdresser, for if he did but know all that is contained in its pages it would go a very long way to help him build up a prosperous business. To our mind, the most interesting chapter by far is the one on the effects of toilet preparations upon the strength of the hair, and, we may add, it is also most instructive. To anyone interested in the growth and care of the hair the little volume will be found of value.

A Handbook for Nurses. By Sydney Welham, M.R.C.S., Resident Medical Officer, Charing Cross Hospital. (London : Mills and Boon, Ltd. Crown 8vo. Pp. 230; with diagrams. 1910. Price 3s. 6d. net.)

In this volume the author has no intention of attempting to teach nursing, which, as he says in his preface, is essentially a practical subject, and only to be acquired by experience in the wards. Its perusal will be no great tax on the understanding of the greenest of probationers, for it is written in clear, simple English, all medical terms employed being carefully explained. Though condensed, the subject-matter is fairly comprehensive, and the information given is accurate. We are glad to see that a chapter has been included on massage and electro-therapeutics, subjects which are at present attracting public attention. A short but useful glossary ends the book. We notice on page 7 that symphysis is spelt symphisis, and we are of opinion that the statement on page 106 that blanc-mange can be given to diabetics is a little sweeping, for some blanc-manges we have met with consisted largely of cornflour.

Dogs, Their Breeds and Characteristics. A popular illustrated review, supervised by A. Knighton, including a dissectible model of a Newfoundland dog. (London and New York : Funk and Wagnalls Co. 1910. Pp. 14, with illustrations in text. Price 7s. 6d.)

After a general survey of the history of the dog through the ages from prehistoric times up to the present day, the common physical characteristics of the *canis familiaris* are dealt with in brief. Each species is then mentioned in detail, differences of coat, height, shape of head and skull, colour and position of eyes, shape of ears, length of neck, shape and size of limbs, shape of nose, and general appearance being given. Each animal is mentioned in its proper place either as a sporting or a non-sporting animal, a small cut of each breed being inserted in the text. The dissectible model of a Newfoundland dog consists of five parts, which show respectively the body, the skeleton, the circulatory organs, the muscular system, and the internal organs and middle section of the body. In this last part the model is so constructed as to exhibit both external and internal appearances of each viscus. Each organ can be so folded to one side as to expose the one beneath. The book and model should prove useful to those who wish to acquire a rough knowledge of the dog's anatomy with a minimum expenditure of labour.

Advice to Consumptives : Home Treatment, After-care, and Prevention. By Noel Dean Bardswell, M.D. (London : Adam and Charles Black, 1910. Crown 8vo. 144 pp. Price 1s. 6d. net.)

While mainly derived from a series of lectures given by the writer from time to time to his patients at the King Edward VII. Sanatorium, this little book has been written with the object of offering advice to those who, having already had the advantage of sanatorium treatment, have returned once more to ordinary home life. It is also hoped that the far larger class who, not being victims of the disease, are desirous of taking all the necessary precautions to avoid tuberculosis, will be successfully appealed to. The advice given throughout the book is sound, and an interesting feature is the inclusion of a chapter on emigration for consumptives. The experiences of former patients who have left England for the colonies and foreign countries usually recommended as suitable to the phthisical are given, and the prospects for each kind of emigrant set forth at length. Would-be emigrants would do well to study this chapter thoroughly before embarking on an expedition which they might afterwards regret. Capital and the possession of health sufficient to do really hard work seem to be absolutely essential for success in practically all colonies. The consumptive who possesses only one or neither of these had better stay at home.

Human Anatomical Model. Being a life-size reproduction of the dissections of the various parts of the human body. (London : The Gresham Publishing Company, 34 and 35 Southampton Street, W.C. Price 45s. net.)

This is an extremely useful and accurate model of dissections, and should prove very suitable for demonstration work in classes where it is impossible to obtain subjects for actual dissections. Lecturers to first-aid voluntary detachments, to nurses, ambulance classes, and school physiology and anatomy courses, will find it serviceable and handy. The female model, which has been submitted to us, is in every way a correct delineation of actual dissections, and although it does not possess the minute finish with which we are acquainted in Brodie's or Zuckerkandl's work, it is nevertheless beautifully executed. Models on the flat necessarily lose something of their value, but in this case the usefulness of the work has been enhanced by a series of plates which gives the side and back views as well as the frontal plane sections, so that it is possible for the student to obtain some idea of the perspective view of parts. The plates show the relations of the various internal organs to one another, as well as the interior of the uterus and its contents at the different stages of pregnancy. The colours used are close copies of the tints seen in a well-injected subject, although the shades have, of course, been strengthened to show up some of the minuter vessels and nerves. The model, which is mounted on strong board, edged with cloth, and which can be packed into a relatively small compass and readily suspended on the wall, is supplied for professional or scientific purposes only, and is not sold to members of the general public. We can thoroughly recommend it to the attention of those who are desirous of possessing a really sound and useful model for demonstration purposes.

